# Improvement of Impact Strength of Polylactide Blends with a Thermoplastic Elastomer Compatibilized with Biobased Maleinized Linseed Oil for Applications in Rigid Packaging

**DOI:** 10.3390/molecules26010240

**Published:** 2021-01-05

**Authors:** Ramon Tejada-Oliveros, Rafael Balart, Juan Ivorra-Martinez, Jaume Gomez-Caturla, Nestor Montanes, Luis Quiles-Carrillo

**Affiliations:** Technological Institute of Materials (ITM), Universitat Politècnica de València (UPV), Plaza Ferrándiz y Carbonell 1, 03801 Alcoy, Spain; rateol@epsa.upv.es (R.T.-O.); juaivmar@doctor.upv.es (J.I.-M.); jaugoca@epsa.upv.es (J.G.-C.); nesmonmu@upvnet.upv.es (N.M.)

**Keywords:** polylactide, thermoplastic elastomer, impact strength, rigid packaging, mechanical properties

## Abstract

This research work reports the potential of maleinized linseed oil (MLO) as biobased compatibilizer in polylactide (PLA) and a thermoplastic elastomer, namely, polystyrene*-b-*(ethylene*-ran-*butylene)*-b-*styrene (SEBS) blends (PLA/SEBS), with improved impact strength for the packaging industry. The effects of MLO are compared with a conventional polystyrene*-b-*poly(ethylene*-ran-*butylene)*-b-*polystyrene*-graft-*maleic anhydride terpolymer (SEBS*-g-*MA) since it is widely used in these blends. Uncompatibilized and compatibilized PLA/SEBS blends can be manufactured by extrusion and then shaped into standard samples for further characterization by mechanical, thermal, morphological, dynamical-mechanical, wetting and colour standard tests. The obtained results indicate that the uncompatibilized PLA/SEBS blend containing 20 wt.% SEBS gives improved toughness (4.8 kJ/m^2^) compared to neat PLA (1.3 kJ/m^2^). Nevertheless, the same blend compatibilized with MLO leads to an increase in impact strength up to 6.1 kJ/m^2^, thus giving evidence of the potential of MLO to compete with other petroleum-derived compatibilizers to obtain tough PLA formulations. MLO also provides increased ductile properties, since neat PLA is a brittle polymer with an elongation at break of 7.4%, while its blend with 20 wt.% SEBS and MLO as compatibilizer offers an elongation at break of 50.2%, much higher than that provided by typical SEBS*-g-*MA compatibilizer (10.1%). MLO provides a slight decrease (about 3 °C lower) in the glass transition temperature (*T_g_*) of the PLA-rich phase, thus showing some plasticization effects. Although MLO addition leads to some yellowing due to its intrinsic yellow colour, this can contribute to serving as a UV light barrier with interesting applications in the packaging industry. Therefore, MLO represents a cost-effective and sustainable solution to the use of conventional petroleum-derived compatibilizers.

## 1. Introduction

In the last decade, a growing social concern about the environment has arisen. This situation is specifically pronounced in the plastics industry due to the huge waste generation, the use of fossil nonrenewable sources and the increasing need to reduce carbon footprint. Currently, polymers and plastics can be found anywhere around us with the subsequent increase in year-by-year production [1,2]. Today, the worldwide plastics production is around 300 Mt per year, and, as most the of the plastics are not biodegradable, they generate a huge amount of plastic waste [3]. This new social awareness is turning the plastics industry towards more environmentally friendly polymeric materials. This transition is focused on two moments related to the life cycle of plastics, i.e., at the origin (natural versus fossil sources) or at the end (potential biodegradation or compost disintegration) [4,5]. In particular, a great rise has been observed in the packaging industry, in which the problem has increased over the years due to massive employment of single-use plastics [6].

To overcome this situation, industry and researchers have been particularly interested in the development of new polymer materials from renewable resources and/or potentially biodegradable (actually, disintegrable in compost soil with specific composition) [7]. For this reason, materials such as aliphatic polyesters, e.g., poly(lactide) (PLA), poly(glycolic acid) (PGA), and others, e.g., poly(hydroxyalkanoates) (PHA) are becoming very popular. These polymers can be obtained from natural resources or by bacterial fermentation, thus leading to high efficiency materials from an environmental point of view [8,9,10,11].

In this sense, PLA is among the most promising biopolymers. PLA can be obtained from starch-rich renewable resources and is increasingly used in the packaging industry, due to its great balance of mechanical, thermal and barrier properties, along with a cost-competitive price and easy processing by conventional techniques [12]. This is why PLA is currently leading the emerging bioplastics market and can be found in a wide range of specialized sectors, such as medical, pharmaceutics and bioengineering, since it is biocompatible and resorbable [13,14]; automotive industry [15,16,17], additive manufacturing and 3d printing [18,19]; or packaging [20,21]. However, PLA is intrinsically very fragile and brittle, and this can represent an important drawback for applications that require somewhat toughness such as 3D-printed parts and rigid packaging [22,23].

To solve (or at least, to minimize) this problem, important research has been carried out in recent years focusing on different approaches to overcome this major drawback for most industrial uses. Many works are focused on the use of plasticizers such as oligomers of lactic acid (OLAs), with exceptional results on toughness [24,25], poly(ethylene glycol) (PEG) [26] or triethyl citrate (TEC) [27], with the main aim of increasing the elongation at break of PLA and its overall ductile behaviour. Nevertheless, the use of plasticizers is usually associated with a decrease in the glass transition temperature (*T_g_*), and subsequently, mechanical resistant properties are lower. Another interesting approach is copolymerization with flexible monomers, for example, poly(lactide)*-g-*poly(butylene succinate*-co-*adipate) [28]. Although a remarkable improvement in toughness can be obtained through copolymerization, this is not a cost-effective solution.

Among the different strategies, the use of binary/ternary blends represents a cost-effective solution to tailor the target properties and to reduce the final cost of biopolymers. To overcome the intrinsic fragility of polymers, these can be blended with flexible polymers that act as impact modifiers. Often, these blends consist of partially miscible or even immiscible flexible polymers that are finely dispersed into the brittle polymer matrix. In these partially or fully immiscible blends, the use of compatibilizers to enhance the interactions between them is encouraged [29]. Poly(styrene)*-b-*(ethylene*-ran-*butylene)*-b-*(styrene) (SEBS) is a thermoplastic elastomer (TPE) that behaves like a rubber and could act as an impact modifier in PLA-based formulations for improved toughness. In general, TPEs offer an excellent balance between processability (thermoplastic) and rubber-like properties, and are increasingly used [30]. SEBS is obtained by hydrogenation of unsaturations contained in polystyrene-butadiene-styrene (SBS). The absence of unsaturations in the hydrogenated structure provides excellent thermal and UV resistance [31], which is a key factor in the food packaging industry. In addition, due to its wide range of elasticity and hardness values (depending on the ethylene-butylene segments) and easy processing at relatively low temperatures, it is used in a wide range of sectors such as adhesives, cable coatings, films, toys, household appliances and, increasingly, in the medical and automotive sectors [32,33]. SEBS has been reported to provide improved impact strength and overall ductility in poly(propylene)/poly(styrene) blends as well as in polyamide-based formulations [34,35,36].

Nevertheless, as above-mentioned, many polymer blends are composed of partially miscible or fully immiscible polymers, which leads to a poor interfacial adhesion. Consequently, the final performance of these blends is lower than expected [37,38]. In these cases, the use of a compatibilizer agent is almost mandatory to provide a bridge or coupling effect between the two polymers in the blend [39]. To improve the miscibility between polymers, there are two main techniques: ex situ (non-reactive) or in situ (reactive) compatibilization. The first technique refers to the use of a third component, usually a grafted or block copolymer obtained under a careful design and synthesis to be largely miscible with both components of the blend. These compatibilizers preferentially are located at the interface between the immiscible (or partially miscible) polymers allowing polymer–polymer interactions which can increase interfacial adhesion and morphological stability, thus improving the overall performance of the blend [40]. Some of these compatibilizers are obtained by grafting a highly reactive group, such as maleic anhydride, carboxylic acid, oxirane ring and acrylate group, into the main polymer backbone to provide dual functionality. Thus, it is possible to find SEBS*-g-*MA which is a SEBS polymer (non-polar) grafted with maleic anhydride (with high polarity). SEBS*-g-*MA is widely used as compatibilizer in polar/non-polar systems due to its dual functionality [41]. A remarkable increase in the impact strength, tensile strength and elongation at break of recycled polyacrylonitrile-butadiene-styrene (rABS) by blending with SEBS and using SEBS*-g-*MA as compatibilizer has been reported [42,43].

The second technique, the so-called reactive extrusion (REX), is a simpler and more cost-effective technique that uses polymers, oligomers and additives that react during the extrusion process with certain functional groups [44]. Usually, REX extrusion requires the use of initiators to introduce a variety of functional groups into biopolymer chains [45]. In recent years, vegetable oils have attracted high interest in the polymer industry as they can be used as renewable sources for polymer synthesis and additives for industrial formulations. Modified vegetable oils have been used as plasticizers, compatibilizers, stabilizers, crosslinkers, among others. It is worth noting the increasing use of epoxidized vegetable oils (EVOS) such as epoxidized soybean oil (ESBO) [46,47], epoxidized linseed oil (ELO) [48,49] or epoxidized palm oil (EPO) [50]. All these EVO have been successfully used in polymer blends and composites with a plasticization/compatibilization main effect, due to the reactivity of the oxirane group. In addition to epoxidized vegetable oils, maleinization is another interesting chemical modification of vegetable oils. Maleinized linseed oil (MLO) has been used as compatibilizer in wood plastic composites with poly(butylene succinate) (PBS) matrix and almond shell flour [51]. The exceptional properties MLO can provide to PLA-based composites with regard to its impact strength and overall ductile properties have been reported [52].

The main objective of this work is to improve the impact strength of PLA by blending with SEBS to increase and broaden the use of PLA in the packaging industry. The novelty of this work is the use of a biobased maleinized linseed oil (MLO) as cost-effective compatibilizer to overcome the immiscibility between both polymers, due to its dual functionality which can interact with both PLA and SEBS. A conventional petroleum-derived SEBS*-g-*MA compatibilizer is used as control compatibilizer since it is widely used in these blends. The effect of these compatibilizing systems on the mechanical, morphological, thermal, thermomechanical, visual aspect and wetting characteristics of PLA/SEBS is studied, and the potential of a biobased compatibilizer derived from linseed oil (MLO) as a potential substitute of other petroleum-derived compatibilizers to provide improved toughness is assessed.

## 2. Results and Discussion

### 2.1. Mechanical Properties

The results of the mechanical characterization of PLA/SEBS blends with different compatibilizing systems are shown in Table 1. These results reveal the effectiveness of each compatibilizing systems used to obtain improved toughness PLA formulations.

**Figure 1:**
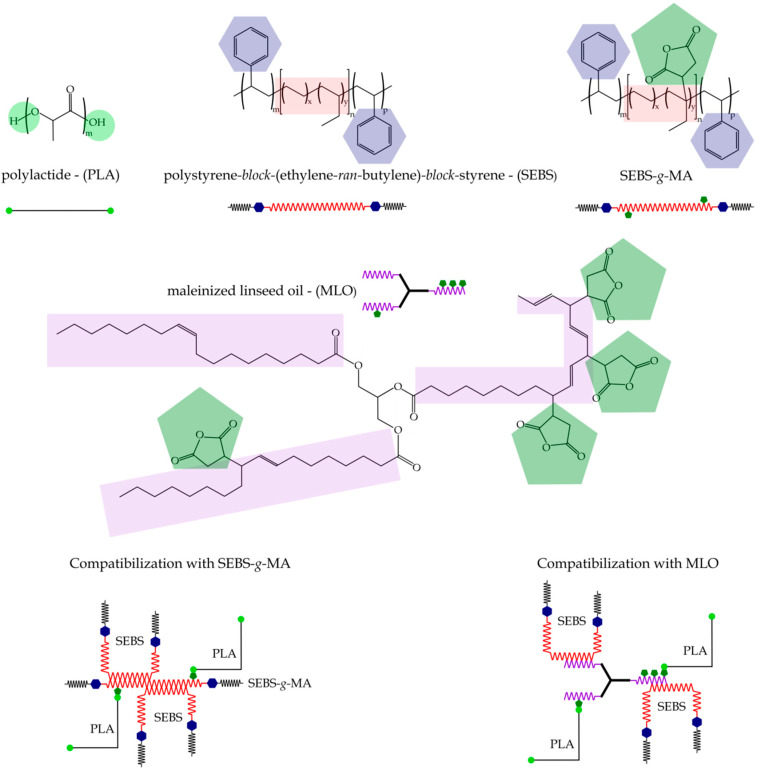
Proposed interaction mechanism between the PLA/SEBS blend and the different compatibilizing agents.

As can be seen, the tensile modulus (E) and the tensile strength (σ_max_) of neat PLA are 2977 and 35.8 MPa, respectively. These mechanical properties are interesting and higher than most commodities; nevertheless, the elongation at break of PLA is only 7.4% which is representative for very low ductile properties, thus leading to a stiff and brittle polymer. These characteristic values of PLA have been reported by other authors [53]. As expected, the addition of SEBS (20 wt.%) leads to a decrease in both tensile modulus and tensile strength. It strikes the low tensile strength values, typical of a rubber-like material, but this can be attributed to the extremely low hardness SEBS grade (5 Shore A). Nevertheless, the elongation at break was slightly improved without any compatibilizer system or, what is more important, it was not dramatically reduced as it happens in immiscible blends. These poor mechanical properties are directly related to the blend structure. It has been reported that PLA and SEBS are not miscible and, consequently, the overall properties of the uncompatibilized PLA/SEBS blend are poor [54]. With the use of different compatibilizing systems on the base PLA/SEBS blend, some interesting findings are revealed. The addition of SEBS*-g-*MA contributes to some compatibilization since the elongation at break is improved, while the stiffness is reduced. This is related to improved PLA/SEBS interactions since the SEBS chains in SEBS*-g-*MA are compatible with SEBS, while the grafted maleic anhydride can react with terminal hydroxyl groups in PLA, thus leading to a coupling effect [55]. MLO-compatibilized PLA/SEBS blend offers interesting properties. The material has a flexible behaviour with a tensile modulus of 1275 MPa and a tensile strength typical of an elastomer (5.2 MPa). It is worth noting the positive effect of MLO on elongation at break of PLA/SEBS blend with a value of 50%. Modified vegetable oils can provide several properties to polyester-type polymers such as chain extension, branching, crosslinking and plasticization. Depending on the main mechanism, different mechanical performance can be obtained. It has been reported that acrylated epoxidized soybean oil (AESBO) shows a remarkable rise in ductile properties of PLA [46]. The compatibilization system that comprises a mixture of SEBS*-g-*MA and MLO gives intermediate values between those offered by the individual compatibilizers. The compatibilizing effects of both maleinized compounds can be schematically seen in Figure 1.

Table 1 also gathers the results corresponding to the impact strength. PLA is a brittle polymer with an impact strength of 1.3 kJ/m^2^. The uncompatibilized PLA/SEBS blend shows a remarkable increase in impact strength up to values of 4.8 kJ/m^2^ which represents a percentage increase of 269%, compared to neat PLA. Lima et al. [54] reported similar values for PLA containing 20% SEBS. This noticeable increase in the impact strength could be attributed to the flexible ethylene-butylene blocks contained in SEBS, which act as impact absorbers due to their rubber-like behaviour. Other studies have revealed the positive effect of SEBS on the impact strength of poly(propylene)-short glass fiber composites. Reactive extrusion with maleic anhydride allowed obtaining an increase of 300% higher than the uncompatibilized composites [56]. Regarding the effect of SEBS*-g-*MA on impact strength, it is worth noting a slight decrease in impact strength with regard to uncompatibilized PLA/SEBS blend. The MLO-based compatibilizer system seems to give the best toughness improvement with an impact strength of 6.1 kJ/m^2^ (almost five times higher than neat PLA), while no synergistic effect can be observed for the binary (SEBS*-g-*MA + MLO) compatibilizing system. By considering both tensile and impact properties, MLO seems to give the best-balanced mechanical properties. The excellent compatibilization/plasticization effect of MLO on binary PLA-thermoplastic starch (TPS) blends has been reported [57].

Regarding Shore D hardness, it is possible to observe the same tendency to that observed for the tensile modulus. Neat PLA has a Shore D hardness values of 80, while its blend with 20 wt.% SEBS shows a Shore D hardness value of 63, thus giving evidence of the effect of the thermoplastic elastomer on hardness. It is worth noting the balanced hardness of MLO-compatibilized PLA/SEBS blends with Shore D values of 58.8.

### 2.2. Morphology of PLA/SEBS Blends

**Figure 2:**
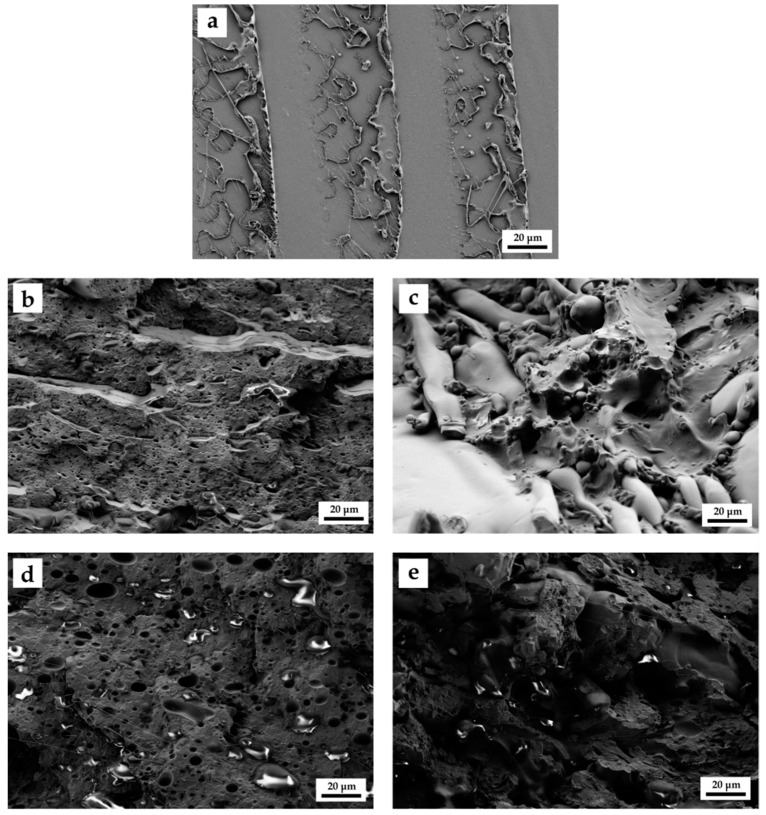
Field emission scanning electron microscopy (FESEM) images at 500× of the fractured surfaces of the different uncompatibilized and compatibilized PLA/SEBS (20 wt.%): (**a**) neat PLA; (**b**) uncompatibilized; (**c**) compatibilized with SEBS*-g-*MA; (**d**) compatibilized with MLO; (**e**) compatibilized with SEBS*-g-*MA/MLO.

Mechanical properties of uncompatibilized and compatibilized PLA/SEBS blends are directly related to their internal structure. Figure 2 gathers the field emission scanning electron microscopy (FESEM) images at 500× of uncompatibilized and compatibilized PLA/SEBS blends after the impact test.

Figure 2a shows the typical fracture surface of neat PLA, with a very flat surface (without plastic deformation) representative of high brittleness. A fractured brittle surface seems to be a polished surface with very low roughness since plastic deformation is not allowed to occur. Fracture starts with some microcracks in micropores and microvoids which can grow in the longitudinal (fracture plane) direction since plastic deformation is restricted. This crack formation-growing process takes place in several parts of the cross-section (the weakest parts) and, since there is very low plastic deformation, fracture occurs leading to a mirror finish surface with evidence of the crack formation and growth, as can be seen in Figure 2a. Immiscibility of the binary PLA/SEBS system is evidenced in Figure 2b in which SEBS spherical or elliptical domains are finely dispersed and embedded into the PLA matrix. This morphology is the typical droplet-like corresponding to immiscible polymer blends. As can be seen, a high number of spherical voids can be detected in Figure 2b, which is attributed to the pulled SEBS particles during the impact test. In addition to the droplet-like morphology, there are poor polymer–polymer interface phenomena which result in poor mechanical properties as shown in Table 1. This poor polymer–polymer interface adhesion does not allow good load transfer and, consequently, the cohesion properties (tensile strength, elongation at break) are not improved in the uncompatibilized PLA/SEBS blend regarding neat PLA; despite this, the impact strength increases due to the rubber-like behaviour of the finely dispersed SEBS microparticles. Nehra et al. [55] reported similar morphologies for PLA/SEBS*-g-*MA blends and the stress concentration effect due to the poor polymer–polymer interactions.

The morphologies of the compatibilized PLA/SEBS blends are remarkably different from those described to date. In general, higher phase continuity can be detected and, subsequently, the number of voids is reduced, while their size is increased. With regard to the MLO-compatibilized systems (Figure 2d), phase separation is still detectable; nevertheless, the spherical voids, representative for the SEBS phase, are more deformed. This could be related to the plasticization effect that MLO provides to the blend, which allows higher plastic deformation. Hence, this morphology is in accordance with the previous mechanical properties. Ferri et al. [57] reported similar morphologies in PLA/TPS blends with different MLO content. Finally, Figure 2e shows the morphology of the mixture with SEBS*-g-*MA and MLO. This mixture gives a break between the MLO and the SEBS*-g-*MA separately, showing a rather rough surface with a good interaction between PLA and SEBS. These results are very much in line with the mechanical properties.

### 2.3. Thermal Properties of PLA/SEBS Blends

With regard to neat PLA, as the used grade is amorphous, which is typically used in films and sheets, it only shows its glass transition temperature (*T_g_*), located at 60–65 °C in a similar way to that described by Kaczmarek et al. [58]. Table 2 gathers the glass transition temperatures (*T_g_*) of the PLA-rich phase obtained by DSC (inflection point). As can be seen, neat PLA is characterized by a *T_g_* of 62.8 °C, and this is slightly reduced by blending it with SEBS without any compatibilizer. This is representative of very restricted compatibility/miscibility between these two polymers, as occurs with other systems such as that reported by Tjong et al. [56] in PP/SEBS composites with short glass fiber. The effect of SEBS*-g-*MA compatibilizer on *T_g_* of PLA-rich phase is almost negligible. It is MLO which gives the lowest *T_g_* compared to the other compatibilizing systems, with a value of 59.8 °C, which could be related to the increase in the free volume that the modified triglyceride structure provides to the blend, thus giving slightly increased chain mobility [52]. This reduction is still very low and indicates poor plasticization effects. Nevertheless, MLO can interact with both polymers in the blend. On one hand, some hydrophobic segments contained in MLO can interact with the ethylene-butylene soft segments, while the grafted maleic anhydride can react with the terminal hydroxyl groups of PLA as shown in Figure 1. Therefore, although MLO can provide different effects to polymer blends and composites, i.e*.,* chain extension, branching, plasticization, compatibilization, crosslinking, among others, in this case, it seems that plasticization is very restricted and, due to the excellent impact strength of the MLO-compatibilized PLA/SEBS blend, it is possible to say that the compatibilization effect is more pronounced than others.

**Figure 3:**
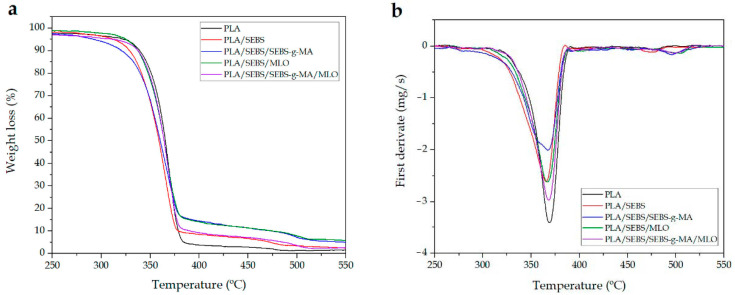
(**a**) Thermogravimetric analysis (TGA) curves and (**b**) first derivative (DTG) of PLA/SEBS blends with different compatibilizers.

The thermal degradation of PLA/SEBS is also slightly changed by using different compatibilizing systems as can be seen in Figure 3 with the corresponding TGA and first derivative (DTG) curves. The main quantitative parameters from the thermal degradation are summarized in Table 3. PLA possesses good thermal stability. In particular, its characteristic temperature for a 5 wt.% mass loss (*T_5%_*) is close to 322 °C, while the maximum degradation rate temperature (*T_deg_*) is 368.9 °C. As reported in other studies, PLA degrades in a single step process with a residual mass of 0.3 wt.% [59]. SEBS addition to PLA leads to a slight decrease in the thermal stability of PLA/SEBS blends. The characteristic *T_5%_* is reduced by almost 10 °C down to 312 °C, while the maximum degradation rate is also lower than neat PLA (365.3 °C). Similar effects of SEBS on thermal degradation have been reported in other systems consisting of poly(propylene) (PP and poly(styrene) (PS) blends as shown by Parameswaranpillai et al. [34].

The most relevant change can be observed by using SEBS*-g-*MA as a compatibilizing system for the base PLA/SEBS blend. The onset degradation temperature, measured as the *T*_5%_, decreases to 291.8 °C, which is 30 °C lower than neat PLA. Chow et al. [60] reported similar behaviour and attributed this decrease in the thermal stability to thermooxidative degradation of ethylene-butylene-styrene segments, triggered in short-length segment hydrocarbons. It is worth noting that SEBS*-g-*MA compatibilized PLA/SEBS blends show a small hump comprised in the 380–500 °C range. This is due to the use of air atmosphere during TGA characterization. As suggested by Chow et al. [60], the maleic anhydride (MA) groups grafted to SEBS can readily react with oxygen at room temperature, thus leading to free radical formation. These free radicals could boost the degradation process and, subsequently, the thermal stability of the PLA/SEBS blends compatibilized with SEBS*-g-*MA is lower.

With regard to the MLO-compatibilized PLA/SEBS blends, it seems that MLO contributes to a slight thermal degradation stabilization or, at least, does not worsen the base values. In fact, the *T*_5%_ is increased by 2 °C with regard to PLA and by 12 °C with regard to the uncompatibilized PLA/SEBS blend. MLO, as other modified vegetable oils, can exert different effects on polymer blends and composites. Some of them are compatibilization and/or crosslinking, which have a positive effect on the thermal stability.

### 2.4. Dynamic-Mechanical Behaviour of PLA/SEBS Blends

**Figure 4:**
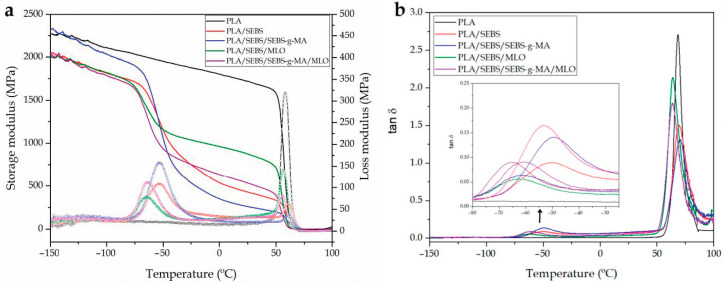
Plot evolution of (**a**) the storage modulus (Line) (*E’*)/Loss modulus (Line + Symbol) (*E*’’) and (**b**) the dynamic damping factor (tan *δ*) of the injection-molded samples of PLA/SEBS blends with different compatibilizers.

Figure 4 gathers the dynamical–mechanical behaviour of neat PLA and uncompatibilized/compatibilized PLA/SEBS blends. Figure 4a shows the plot evolution of the storage modulus (*E’*) and loss modulus (*E*”) as a function of temperature. Neat PLA shows the typical behaviour of a rigid polymer, with a *E’* value of 2100 MPa at −100 °C, which is dramatically reduced down to 35 MPa at 100 °C (above its *T_g_*). At about 70 °C, it is possible to observe a dramatic drop in the storage modulus, which is attributed to the *α*-relaxation process of PLA, or its glass transition temperature (*T_g_*). These characteristic values obtained for PLA agree with other DMTA properties of PLA reported in literature with a *T_g_* in the 60–70 °C range and a storage modulus, *E’* of 1500–2200 MPa, depending on the PLA grade [61,62]. Furthermore, in relation to the *tan δ* values, Lascano et al. [63] showed very similar results to those obtained in this work.

The addition of SEBS provides a remarkable change in the stiffness, as previously described regarding mechanical properties. As can be seen in Figure 4a, the DMTA curve for the uncompatibilized PLA/SEBS blends offers two main drops in the storage modulus. The first one is located at about −50 °C and is directly related to the glass transition temperature, *T_g_* of SEBS. This drop is responsible for the low storage modulus (low rigidity) values at room temperature for all PLA/SEBS blends, as observed previously on tensile properties. The second relevant drop in the storage modulus is located at about 60–70 °C and is related to the glass transition temperature of the PLA-rich phase. Guo et al. [64] also reported a significant decrease in poly(propylene) stiffness by adding SEBS, which is in accordance with the results obtained herein. On the other hand, there is not a remarkable shift in the characteristic *T_g_* values of the PLA- and SEBS-rich phases, thus giving evidence of the immiscibility of both polymers. As Figure 4a suggests, PLA and SEBS offer very restricted miscibility since the corresponding *T_g_* values do not change in a noticeable way, as observed in Table 4, with some mechanical properties obtained by DMTA. By taking the *T_g_* at the maximum value of *tan δ* (peak maximum criterion), the *T_g_* of the PLA-rich phase is 68.5 °C, while this PLA-rich phase shows a *T_g_* of 69.3 °C in the uncompatibilized blend. Nevertheless, the use of maleinized SEBS (SEBS*-g-*MA) has been reported to provide a slight decrease in the *T_g_* of PLA-rich phase. Nehra et al. [55] reported a decrease in the *T_g_* of PLA from 82.4 to 74.1 °C (with the *tan δ* peak maximum criterium). Nevertheless, they used 20 wt.% SEBS*-g-*MA in the blends with PLA. In this work, the amount of SEBS*-g-*MA is noticeably lower and, subsequently, it does not provide a remarkable change in the *T_g_* of the PLA-rich phase as can be seen in Table 4 with values close to 70 °C. As observed in previous techniques, it is MLO which provides a slight decrease in the characteristic *T_g_* value of PLA-rich phase down to values of 63.8 °C. This decrease was also observed by DSC characterization and can be ascribed to a slight plasticization effect of MLO, thus confirming that PLA provides a slight increase in chain mobility whit the subsequent decrease in *T_g_* [65]. Another striking issue is that the storage modulus of all compositions is maintained at high values at −100 °C (below both *T_g_* corresponding to the PLA-rich and SEBS-rich phases). Nevertheless, at room temperature (above the *T_g_* of the SEBS-rich phase and below the *T_g_* of the PLA-rich phase), the differences are much higher due to the softening of the elastomer component in the blend and are in total agreement with the tensile modulus obtained by tensile tests as above-mentioned. Similar behaviour has been reported in immiscible PE/PLA blends with different reactive compatibilizers [66].

### 2.5. Colour Measurement and Wetting Properties of PLA/SEBS Blends

**Figure 5:**
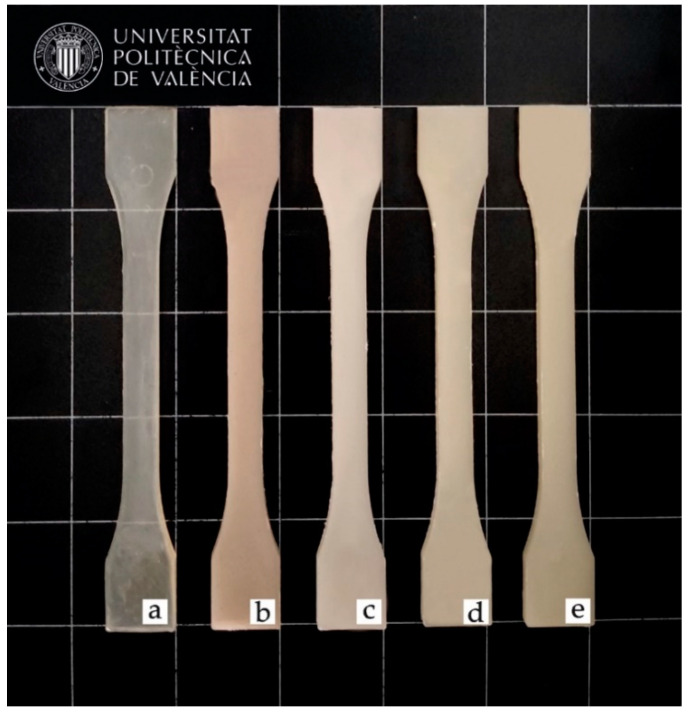
Visual appearance of the samples: (**a**) neat PLA; (**b**) uncompatibilized PLA/SEBS; (**c**) PLA/SEBS compatibilized with SEBS*-g-*MA; (**d**) PLA/SEBS compatibilized with MLO; (**e**) PLA/SEBS compatibilized with SEBS*-g-*MA and MLO.

Colour, luminance and transparency are essential issues to be considered in packaging, as the consumer’s impression of the product depends on them. Table 5 shows the values of the colour coordinates of PLA/SEBS blends, while Figure 5 shows the visual appearance of the tensile test specimens. Neat PLA exhibits clear transparency due to its amorphous structure as confirmed by DSC [67]. Moreover, when using the different compatibilization systems, transparency is lost, probably due the lack of miscibility between both polymers [67].

Colour coordinates L*a*b* were measured on injection-moulded samples. Luminance (L*) refers to the clarity or lightness. There seems to be little change in the studied samples, although the addition of compatibilizers in PLA/SEBS blends provides slight yellowing, especially in cases where MLO is used as a compatibilizing system. Regarding the a* colour coordinate, it indicates the colour change between green (negative) and red (positive). All samples show negative values, except that corresponding to the uncompatibilized PLA/SEBS blend, which shows a slight yellow-to-brown colour. All registered a* values are close to 0, due to the white shade of the samples, although it can be seen as the addition of compatibilizers that brings those values near to the negative region, and therefore, towards green shades, especially in samples with MLO. Quiles-Carrillo et al. [68] observed a similar change in colour in PA1010-based materials with MLO. Concerning the b* coordinate, it is indicative of the yellow (positive) and blue (negative) region. All studied materials exhibit positive values, due to their yellowish-white colour, and brown in the case of PLA/SEBS. All used compatibilizers increase the b* coordinate, thus indicating some yellowing. This is particularly pronounced in MLO-compatibilized PLA/SEBS blends, with b* values close to 10, which is consistent with the visual aspect. It is important to bear in mind the intrinsic yellow colour of MLO. This deviation towards more yellow tones is directly reflected in the yellowness index shown in Table 5. Yellowness is associated with an increase in chromophore groups, whose conjugation causes absorption at higher wavelengths. In particular, the blends containing MLO cause the greatest yellowing in the samples. This index has a great advantage from the point of view of the packaging of certain elements, allowing better protection against light [69].

On the one hand, the opacity and slight yellow colour of PLA/SEBS blends could represent a disadvantage in certain packaging sectors, where transparency is required, but on the other hand, they could be an advantage when it comes to preventing UV radiation from harming sensitive products, as could be the case for food and pharmaceutical industries [70]. So, opaque PLA/SEBS blends can act as a barrier against food degradation provoked by UV radiation. Barreto et al. reported the effectiveness of SEBS as a protective agent against UV radiation when used as compatibilizer [71].

**Figure 6:**
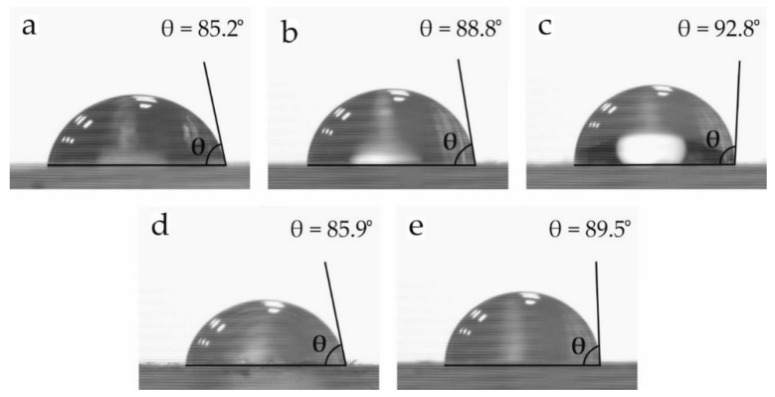
Water contact angle of the samples: (**a**) neat PLA; (**b**) uncompatibilized PLA/SEBS; (**c**) PLA/SEBS compatibilized with SEBS*-g-*MA; (**d**) PLA/SEBS compatibilized with MLO; (**e**) PLA/SEBS compatibilized with SEBS*-g-*MA and MLO.

The water contact angle (*θ_w_*) of neat PLA and PLA/SEBS blends with different compatibilizers was obtained to evaluate their wetting properties. A high *θ_w_* is representative of poor surface affinity to water. As can be seen in Figure 6, all samples present contact angles far superior to 65°, which could be considered the hydrophobicity threshold [72]. Although PLA has some polar groups in its structure, it is a hydrophobic polymer with a *θ_w_* of 85.2°. The addition of SEBS and SEBS*-g-*MA slightly increases the hydrophobicity of PLA up to values of 88.8° and 92.8°, respectively. It is worth noting that SEBS is a highly hydrophobic terpolymer, as Fang et al. reported [73]. With regard to MLO, it does not provide a remarkable change in the wetting properties, while the combined effect of MLO and SEBS*-g-*MA leads to a slightly higher *θ_w_* of 89.5°.

## 3. Materials and Methods

### 3.1. Materials

The base poly(lactic acid) was an Ingeo^TM^ 2003D commercial grade, supplied by Natureworks (Minnetonka, MN, USA). This PLA grade has a density of 1.24 g/cm^3^ and a melt flow index (MFI) of 6 g/10 min (measured at 210 °C and a load of 2.16 kg). This PLA grade offers high transparency and finds applications in the food-packaging sector. The impact modifier was a polystyrene*-b-*poly(ethylene*-ran-*butylene)*-b-*polystyrene terpolymer, SEBS, with an extremely low hardness (Shore A hardness = 5) grade Megol TA 5^®^ Neutral. This was supplied by Applicazioni Plastiche Industriali (Api SpA, Vicenza, Italy) and has a density of 0.88–0.89 g/cm^3^. It has an extremely low tensile modulus (1.1–4.2 MPa at 100% elongation) and low tensile strength (<6 MPa).

**Figure 7:**
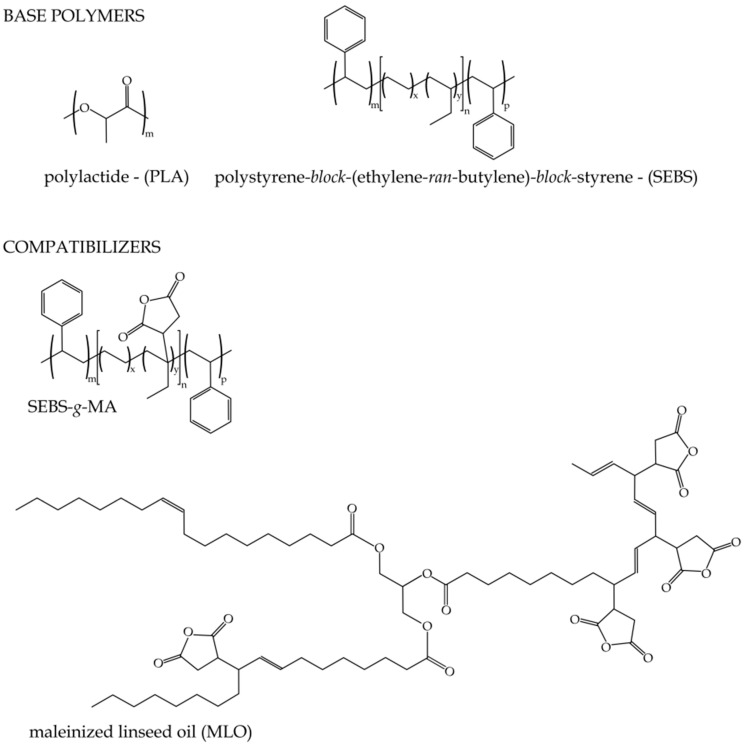
Chemical structure of the base polymer and the different compatibilizers used in this work.

Different compatibilizers with grafted maleic anhydride were selected to improve the toughness of PLA/SEBS blends. One of them was a polystyrene*-b-*poly(ethylene*-ran-*butylene)*-b-*polystyrene*-graft-*maleic anhydride terpolymer (SEBS*-g-*MA) with CAS number 124578-11-6 and a melt index of 21 g/10 min (230 °C/5.0 kg) and was supplied by Sigma Aldrich S.A. (Madrid, Spain). An environmentally friendly maleinized linseed oil (MLO), VEOMER LIN, was supplied by Vandeputte (Mouscron, Belgium). This modified vegetable oil possesses a viscosity of 10 dPa s at 20 °C and an acid value of 105–130 mg KOH/g. Figure 7 shows the chemical structure of the different polymers and compatibilizers used in this work.

### 3.2. Preparation of PLA/SEBS Blends

PLA and SEBS were initially dried at 40 °C for 48 h in a dehumidifying dryer MDEO to remove any residual moisture prior to processing. Then, the corresponding wt.% of each component (see Table 6) was mixed and pre-homogenized in a zipper bag.

The corresponding formulations were compounded in a twin-screw extruder from Construcciones Mecánicas Dupra, S.L. (Alicante, Spain). This extruder has a 25 mm diameter with a length-to-diameter ratio (L/D) of 24. The extrusion process was carried out at a rate of 18 rpm, using the following temperature profile (from the hopper to the die): 175–180–190–195 °C. The compounded materials were pelletized using an air-knife unit. In all cases, residence time was approximately 1 min. Table 6 shows the compositions of the materials developed in this work.

To transform the pellets into standard samples, a Meteor 270/75 injection moulding machine from Mateu & Solé (Barcelona, Spain) was used. The temperature profile in the injection moulding unit was 175 (hopper), 180, 190 and 195 °C (injection nozzle). A clamping force of 75 tons was applied, while the cavity filling and cooling times were set to 1 and 10 s, respectively. Standard samples for mechanical and thermal characterization with an average thickness of 4 mm were obtained.

### 3.3. Characterization of PLA/SEBS Blends

#### 3.3.1. Mechanical Characterization

Tensile properties of PLA/SEBS blends were obtained in a universal testing machine ELIB 50 from S.A.E. Ibertest (Madrid, Spain) as recommended by ISO 527-1:2012. A 5-kN load cell was used and the cross-head speed was set to 5 mm/min. Shore hardness was measured in a 676-D durometer from J. Bot Instruments (Barcelona, Spain), using the D-scale, on rectangular samples with dimensions 80 × 10 × 4 mm^3^, according to ISO 868:2003. The impact strength was also studied on injection-moulded rectangular samples with dimensions of 80 × 10 × 4 mm^3^ in a Charpy pendulum (1-J) from Metrotec S.A. (San Sebastián, Spain) on notched samples (0.25 mm radius V-notch), following the specifications of ISO 179-1:2010. All mechanical characterizations were performed at room temperature, and a minimum of 6 specimens of each blend formulation were tested, and the characteristic values were averaged. For tensile tests, the tensile modulus (*E*), the percentage elongation at break (%*ε_b_*) and the maximum tensile strength (*σ_max_*) were obtained. The impact strength was obtained from Charpy tests, and the Shore D hardness average values were obtained using the Shore D durometer.

#### 3.3.2. Morphology Characterization

The morphology of the fractured samples from impact tests was studied by field emission scanning electron microscopy (FESEM) in a ZEISS ULTRA 55 microscope from Oxford Instruments (Abingdon, United Kingdom). Before placing the samples in the vacuum chamber, they were sputtered with a gold-palladium alloy in an EMITECH sputter coating SC7620 model from Quorum Technologies, Ltd. (East Sussex, UK). The FESEM was operated at an acceleration voltage of 2 kV.

#### 3.3.3. Thermal Analysis

The most relevant thermal transitions of PLA/SEBS blends were obtained by differential scanning calorimetry (DSC) in a Mettler-Toledo 821 calorimeter (Schwerzenbach, Switzerland). Small rectangular-like samples with an approximate side length of 1–2 mm, with an average weight of 5–7 mg, were subjected to a thermal program divided into three stages: a first heating from 25 to 170 °C followed by a cooling to 0 °C, and a second heating to 250 °C. Both heating and cooling rates were set to 10 °C/min. All tests were run in nitrogen atmosphere with a flowrate of 66 mL/min using standard sealed aluminium crucibles with a capacity of 40 μL.

The thermal degradation of the PLA/SEBS blends was assessed by thermogravimetric analysis (TGA). TGA tests were performed in a LINSEIS TGA 1000 (Selb, Germany). Samples with a weight of 5–7.5 mg were placed in 70 µl alumina crucibles and subjected to a dynamic heating program from 30 to 700 °C at a heating rate of 10 °C/min in air atmosphere. The first derivative thermogravimetric (DTG) curves were also determined. All tests were carried out in triplicate to obtain reliable results, and the provided curves and thermograms correspond to the average of these three tests.

#### 3.3.4. Dynamical–Mechanical Thermal Characterization

Dynamical–mechanical thermal analysis (DMTA) was carried out in a DMA1 dynamic analyzer from Mettler-Toledo (Schwerzenbach, Switzerland), working in single cantilever flexural conditions. Rectangular samples with dimensions 20 × 6 × 2.7 mm^3^ were subjected to a dynamic temperature sweep from −150 to 120 °C at a constant heating rate of 2 °C/min. The selected frequency was 1 Hz, and the maximum flexural deformation or cantilever deflection was set to 10 µm. DMTA tests were run in triplicate and averaged.

#### 3.3.5. Colour and Wetting Characterization

A Konica CM-3600d Colorflex-DIFF2, from Hunter Associates Laboratory, Inc. (Reston, Virginia, USA) was used for the colour measurement. Colour coordinates (L*a*b*) were measured according to the following criteria: L* = 0, darkness; L* = 100, lightness; a* represents the green (a* < 0) to red (a* > 0); b* stands for the blue (b* < 0) to yellow (b* > 0) coordinate. The yellowness index for each sample was calculated according to ASTM E313. Colour measurements were carried out on standard tensile samples and, at least five different measurements of the colour coordinates were obtained and averaged.

Contact angle measurements were carried out with an EasyDrop Standard goniometer model FM140 (KRÜSS GmbH, Hamburg, Deutschland) which is equipped with a video capture kit and analysis software (Drop Shape Analysis SW21; DSA1). Double distilled water was used as test liquid. The wetting properties were evaluated on the surface of rectangular 80 × 10 × 4 mm^3^ samples. At least 10 measurements of the water contact angle were collected and averaged.

#### 3.3.6. Statistical Analysis

The mechanical properties were evaluated through analysis of variance (ANOVA) using STATGRAPHICS Centurion XVI v 16.1.03 from StatPoint Technologies, Inc. (Warrenton, VA, USA). Fisher’s least significant difference (LSD) was used at the 95% confidence level (*p* < 0.05). Mean values and standard deviations were also reported.

## 4. Conclusions

This work reports the development of polylactide (PLA) formulations with improved toughness by blending with a thermoplastic elastomer, namely, polystyrene*-b-*(ethylene*-ran-*butylene)*-b-*styrene (SEBS) polymer. PLA/SEBS blends with 20 wt.% SEBS can be processed by extrusion and subsequent injection moulding. The rubber-like nature of SEBS provides improved toughness to PLA. Neat PLA is a brittle material with an impact strength (Charpy) of 1.3 kJ/m^2^, while its blend with 20 wt.% SEBS shows a remarkable increase in toughness with an impact strength of 4.8 kJ/m^2^. Despite this, PLA/SEBS blends are immiscible as observed by field emission scanning electron microscopy (FESEM) and thermal analysis characterization. To improve the impact strength, two different maleinized compounds were used: one petroleum-derived compound polystyrene*-b-*(ethylene*-ran-*butylene)*-b-*styrene*-graft-*maleic anhydride (SEBS*-g-*MA) and one biobased maleinized triglyceride from linseed, namely, maleinized linseed oil (MLO). The best results in terms of impact strength were obtained with 5 phr MLO addition with an impact strength of 6.1 kJ/m^2^. Moreover, MLO also contributes to increasing the low elongation at break of neat PLA from 7.4% up to values of 50.2%, thus showing the efficiency of this compatibilizer. In addition, MLO also provides slightly improved thermal stability, as thermogravimetric characterization has revealed. Dynamic mechanical thermal analysis (DMTA) also corroborated poor miscibility between PLA and SEBS since the corresponding *T_g_* of the PLA- and SEBS-rich phases remains almost invariable. The morphology of the PLA/SEBS blends was highly dependent on the compatibilizer system. While PLA offers a typical brittle flat surface, PLA/SEBS blends offer the characteristic immiscible morphology with a brittle PLA matrix in which SEBS microspheres (droplet like) are finely dispersed. The use of MLO leads to a change in the spherical SEBS domains to deformed shapes, thus indicating increased ductility properties. Overall, the results obtained in this work offer a cost-effective solution to partially overcome the intrinsic brittleness of PLA, thus broadening its potential applications in the packaging industry.

## Figures and Tables

**Table 1:** Summary of mechanical properties of the injection-molded samples of uncompatibilized and compatibilized PLA/SEBS blends. Tensile modulus (*E*), maximum tensile strength (*σ_max_*) and elongation at break (%*ε_b_*), Shore D hardness and impact (Charpy) strength.

Code	E (MPa)	σ_max_ (MPa)	ε_b_ (%)	Shore D Hardness	Impact Strength (kJ/m^2^)
PLA	2977 ± 27 ^a^	35.8 ± 2.6 ^a^	7.4 ± 0.8 ^a^	80.0 ± 0.8 ^a^	1.3 ± 0.1 ^a^
PLA/SEBS	1419 ± 32 ^b^	6.3 ± 0.7 ^b^	7.8 ± 1.0 ^a^	63.0 ± 0.7 ^b^	4.8 ± 0.5 ^b^
PLA/SEBS/SEBS*-g-*MA	802 ± 39 ^c^	4.7 ± 0.6 ^c^	10.1 ± 1.1 ^b^	51.6 ± 1.1 ^c^	4.0 ± 0.1 ^b^
PLA/SEBS/MLO	1275 ± 61 ^b^	5.2 ± 0.6 ^c^	50.2 ± 4.3 ^c^	58.8 ± 0.4 ^b^	6.1 ± 0.6 ^c^
PLA/SEBS/SEBS*-g-*MA/MLO	897 ± 83 ^c^	5.9 ± 0.6 ^b^	23.0 ± 3.0 ^d^	58.2 ± 0.8 ^b^	3.5 ± 0.1 ^b^

^a–d^ Different letters in the same column indicate a significant difference among the samples (*p* < 0.05).

**Table 2:** Glass transition temperature (*T_g_*) of the PLA-rich phase in PLA/SEBS blends with different compatibilizers, obtained by differential scanning calorimetry (DSC).

Code	*T_g_* (°C)
PLA	62.8 ± 0.5
PLA/SEBS	61.9 ± 0.3
PLA/SEBS/SEBS*-g-*MA	61.8 ± 0.4
PLA/SEBS/MLO	59.8 ± 0.2
PLA/SEBS/SEBS*-g-*MA-MLO	60.9 ± 0.1

**Table 3:** Main thermal degradation parameters of the PLA/SEBS blends with different compatibilizers in terms of the onset degradation temperature at a mass loss of 5 wt.% (*T*_5%_), maximum degradation rate (peak) temperature (*T_deg_*) and residual mass at 600 °C.

Code	*T*_5%_ (°C)	*T_deg_* (°C)	Residual Weight (%)
PLA	322.8 ± 1.5	368.9 ± 2.2	0.3 ± 0.1
PLA/SEBS	312.3 ± 1.2	365.3 ± 1.7	0.4 ± 0.1
PLA/SEBS/SEBS-g-MA	291.8 ± 1.1	367.1 ± 1.5	0.6 ± 0.2
PLA/SEBS/MLO	324.8 ± 1.5	366.8 ± 1.8	0.3 ± 0.2
PLA/SEBS/SEBS-g-MA/MLO	312.4 ± 1.1	368.1 ± 0.9	0.2 ± 0.1

**Table 4:** Dynamic-mechanical properties of injection-moulded samples of PLA/SEBS blends with different compatibilizers, at different temperatures.

Parts	*E’* (MPa) at −100 °C	*E’* (MPa) at 25 °C	*E’* (MPa) at 100 °C	*T_g PLA_* (°C) *
PLA	2110 ± 28	1720 ± 14	35.3 ± 1.1	68.5 ± 0.8
PLA/SEBS	1815 ± 17	427 ± 8	4.9 ± 0.3	69.3 ± 0.9
PLA/SEBS/SEBS-g-MA	2030 ± 30	260 ± 7	3.1 ± 0.2	70.0 ± 0.8
PLA/SEBS/MLO	1780 ± 17	875 ± 10	5.9 ± 0.6	63.8 ± 0.8
PLA/SEBS/SEBS-g-MA/MLO	1780 ± 25	265± 12	8.1 ± 0.3	63.7 ± 1.1

* The *T_g_* was measured using the *tan δ* peak maximum criterion.

**Table 5:** Luminance and colour coordinates (L*a*b*) of the PLA/SEBS blends with different compatibilizers.

Code	L*	a*	b*	Yellowness Index (YI)
PLA	40.7 ± 0.3	−0.45 ± 0.03	4.71 ± 0.13	21.3 ± 0.3
PLA/SEBS	53.2 ± 0.1	1.42 ± 0.01	6.76 ± 0.06	26.3 ± 0.1
PLA/SEBS/SEBS-g-MA	59.3 ± 0.1	−0.82 ± 0.02	5.57 ± 0.10	19.3 ± 0.1
PLA/SEBS/MLO	58.4 ± 0.3	−2.36 ± 0.02	8.69 ± 0.18	25.2 ± 0.3
PLA/SEBS/SEBS-g-MA-MLO	61.1 ± 0.1	−1.74 ± 0.01	9.94 ± 0.02	28.1 ± 0.1

**Table 6:** Summary of compositions according to the weight content (wt.%) of PLA/SEBS and different compatibilizers.

Code	PLA (wt.%)	SEBS (wt.%)	SEBS-g-MA (wt.%)	MLO (phr)
PLA	100	0	0	0
PLA/SEBS	80	20	0	0
PLA/SEBS/SEBS*-g-*MA	80	18	2	0
PLA/SEBS/MLO	80	20	0	5
PLA/SEBS/SEBS*-g-*MA/MLO	80	19	1	2.5

## Data Availability

The data presented in this study are available in this article.
